# Cotton aphid infestation monitoring using Sentinel-2 MSI imagery coupled with derivative of ratio spectroscopy and random forest algorithm

**DOI:** 10.3389/fpls.2022.1029529

**Published:** 2022-11-29

**Authors:** Hancong Fu, Hengqian Zhao, Rui Song, Yifeng Yang, Zihan Li, Shijia Zhang

**Affiliations:** ^1^ College of Geoscience and Surveying Engineering, China University of Mining and Technology (Beijing), Beijing, China; ^2^ State Key Laboratory of Coal Resources and Safe Mining, China University of Mining and Technology (Beijing), Beijing, China

**Keywords:** cotton aphid infestation, Sentinel-2 image, derivative of ratio spectroscopy, random forest, Pearson correlation analysis

## Abstract

Aphids are one of the main pests of cotton and have been an important disaster limiting cotton yield. It is important to use satellite multispectral data to monitor the severity of cotton aphids in a timely and accurate manner on regional scale. Based on the combination of derivative of ratio spectra (DRS) and random forest (RF) algorithm, this study researched the quantitative monitoring model of cotton aphid severity based on Sentinel-2 data. First, the cotton area was extracted by using a supervised classification algorithm and the vegetation index threshold method. Then, the DRS algorithm was used to analyze the spectral characteristics of cotton aphids from three scales, and the Pearson correlation analysis algorithm was used to extract the bands significantly related to aphid infestation. Finally, the RF model was trained by ground sampling points and its accuracy was evaluated. The optimal model results were selected by the cross-validation method, and the accuracy was compared with the four classical classification algorithms. The results showed that (1) the canopy spectral reflectance curves at different grades of cotton aphid infestation were significantly different, with a significant positive correlation between cotton aphid grade and spectral reflectance in the visible band range and a negative correlation in the near-infrared band range; (2) The DRS algorithm could effectively remove the interference of the background endmember of satellite multispectral image pixels and enhance the aphid spectral features. The analysis results from three different scales and the evaluation results demonstrate the effectiveness of the algorithm in processing satellite multispectral data; (3) After the DRS processing, Sentinel-2 multispectral images could effectively classify the severity of cotton aphid infestation by the RF model with an overall classification accuracy of 80% and a kappa coefficient of 0.73. Compared with the results of four classical classification algorithms, the proposed algorithm has the best accuracy, which proves the superiority of RF. Based on satellite multispectral data, the DRS and RF can be combined to monitor the severity of cotton aphids on a regional scale, and the accuracy can meet the actual need.

## 1 Introduction

Cotton aphid (*Aphis gossypii* L.) has always been one of the important disasters limiting cotton yield. It has the characteristics of large-scale outbreaks and is widely distributed in major cotton regions all over the world (Ali et al., 2016; Allen et al., 2018; Chen et al., 2018);. At present, aphis gossypii is still mainly controlled by pesticides, but blindly spraying pesticides not only causes waste of resources but also causes serious pollution to the ecological environment (Lu et al., 2012; Xie et al., 2019; Heydari et al., 2020; Alengebawy et al., 2021). Therefore, timely and accurate monitoring of the location and severity of aphis gossypii is important for its precise control (Zhang et al., 2017; Lou et al., 2018; Lin et al., 2021).

Traditional crop pest monitoring methods are mainly based on manual detection, although the accuracy is high, it is usually labor intensive. It is time-consuming and laborious in large-scale monitoring, and there is an inevitable time lag and strong subjectivity, which cannot meet the need for accurate pesticide application. Remote sensing technology has the advantages of timely, effective, synchronous and rapid acquisition, and can obtain continuous surface information, which has become an important tool in the field of crop pest monitoring (Yuan et al., 2017; Yang, 2020; Zhao et al., 2020).

At present, some scholars have studied the growth of cotton under stress at the leaf scale and canopy scale by using near-ground hyperspectral and UAV hyperspectral data, and the results show that the spectral characteristics of cotton under different stress grades have changed, indicating that hyperspectral remote sensing has great application potential in crop monitoring (Nagasubramanian et al., 2018; Yu et al., 2018a; Guo et al., 2021). Hyperspectral remote sensing has been widely used in small and medium-scale crop detection (Liu et al., 2020; Terentev et al., 2022). However, due to the difficulty of obtaining hyperspectral data, there are still deficiencies in large-scale crop monitoring. Therefore, regional scale monitoring by satellite multispectral images is also the current research trend (Su et al., 2018; Zhang et al., 2019).

In recent years, the multispectral satellite sensor represented by Sentinel-2 has provided users with remote sensing data of higher spectral resolution and large-scale spatial range, which has great potential for the quantitative analysis of cotton pests (Liu et al., 2018; Zheng et al., 2018; Li et al., 2020). However, due to the limitation of spatial resolution, the cotton pest information contained in satellite multispectral data is the result of the comprehensive action of two factors, the abundance of cotton pests and the pest degree. Therefore, traditional qualitative analysis is difficult to meet the accuracy requirements of cotton pest monitoring. At present, it is still a challenge to eliminate the interference of factors other than pest information in the spectral feature extraction of remote sensing images. The derivative of ratio spectroscopy (DRS) algorithm is an innovative spectral processing algorithm, which can effectively eliminate the influence of the background spectrum from the mixed pixels, enhance spectral contrast and improve the extraction accuracy of target feature information. It has been applied in many fields and has achieved good results (Philpot, 1991; Zhao et al., 2013; Guo et al., 2021). The random forest (RF) algorithm is an integrated machine learning algorithm, which uses multiple decision trees as the basic classifier to discriminate and classify, and selects different training samples and different features to improve the accuracy and generalization performance of the model, which has the advantages of fast speed, strong anti-noise, and ability to process big data, and has been widely used in the field of remote sensing image classification (Yu et al., 2018b; Zhang et al., 2020).

In this study, ground-truthed hyperspectral data combined with Sentinel-2 satellite multispectral data were used to remove healthy cotton spectra by the DRS algorithm, and the spectral characteristics of different grades of cotton aphid infestation were studied at three different scales. The sensitive bands of aphid infestation were extracted by the Pearson correlation analysis method, and a quantitative analysis model of cotton aphid infestation based on Sentinel-2 data was established based on the RF algorithm, which was used to enhance the application capability of satellite multispectral data in cotton pest monitoring and precision agriculture.

The main work of this study is as follows: (1) Based on the multitemporal Sentinel-2 satellite data, the cotton area in the study area was extracted by using the supervised classification algorithm and vegetation index (VI) threshold method to remove unnecessary disturbing factors for the classification of aphid infestation severity; (2) The DRS algorithm was used to process and analyze the ground hyperspectral data, ground hyperspectral resampling data and Sentinel-2 multispectral image sampling point data. The sensitive bands associated with aphid infestation were extracted by Pearson correlation analysis, and the effectiveness of the DRS algorithm was verified; (3) The RF algorithm was trained and accuracy validated by multispectral image sample data for Sentinel-2 image classification and compared with four classification algorithms, spectral angle matching (SAM) (Kruse et al., 1993), support vector machine (SVM) (Pujari et al., 2016), decision tree (DT) (Marin et al., 2021) and BP neural network (Xu et al., 2020).

## 2 Materials and methods

### 2.1 Experimental study and materials

**Figure 1:**
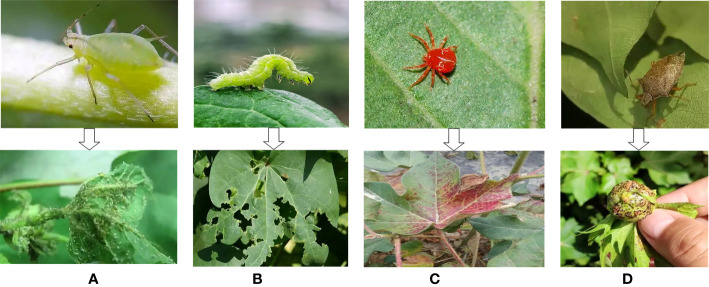
The main pests of cotton in Xinjiang and their harm to cotton. **(A)** Cotton aphid, **(B)** cotton bollworm, **(C)** cotton red spider, **(D)** cotton plant bug.

Xinjiang has very superior climate resources for agricultural production, and is especially suitable for cotton cultivation. Xinjiang is currently the main cotton growing area in China, accounting for 78.9% of the total cotton growing area and 87.3% of the total cotton production in the country in 2020. Due to the long growth period of cotton, the pest infestation effect is very obvious, resulting in many yield losses. There are four main species of cotton pests in Xinjiang, namely cotton aphid, cotton bollworm, cotton red spider and cotton plant bug (as shown in **Figure 1**). The cotton aphid cause leaves to shrink and secrete honeydew on leaves, which can easily cause the leaves to wither and fall off, affecting the cotton quality. The impact of cotton bollworms on cotton is mainly to eat leaves, flowers, and also to arch the boll. The cotton red spider causes the entire cotton plant to redden and dry out, resulting in the loss of flowers and bolls. The cotton plant bug causes black spots on plants that lose their green color sucking the growing points of the cotton as well as causing the bolls to die or fall off. Among them, the cotton aphid is a long-lasting, heavy and fast breeder, prone to drug resistance and is harmful throughout the cotton reproductive process, especially at the seedling and boll stages. In this study, the cotton aphid was chosen as the research object.

**Figure 2:**
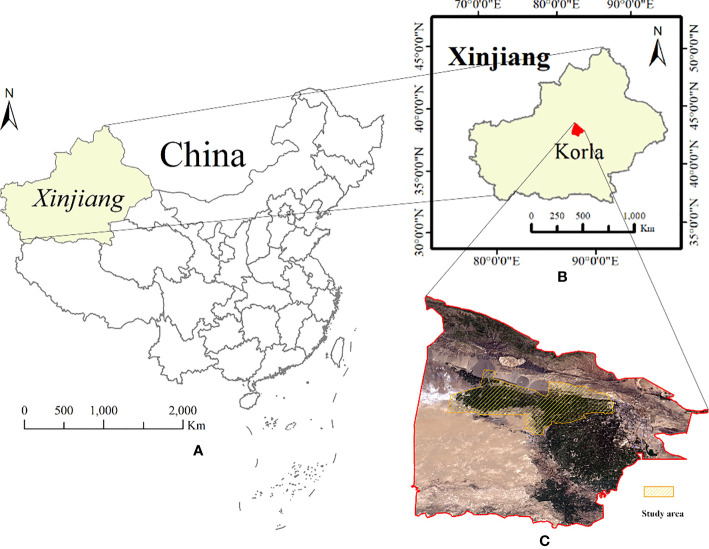
Location of the study area and Sentinel-2 imagery. **(A)** China map, **(B)** Korla city, Bayingolin Mongolian Autonomous Prefecture, Xinjiang, **(C)** Sentinel-2 satellite multispectral image of the study area, note, Red: B4 band; Green: B3 band; Blue: B2 band.

Korla is the capital of Bayingolin Mongolian Autonomous Prefecture in Xinjiang, with east longitude 85°14′10″-86°34′21″ and north latitude 41°10′48″-42°21′36″. It is located in the central part of Xinjiang, the southern foot of Tianshan Mountain, the northeast edge of the Tarim Basin, south of the world’s second largest desert - Taklimakan desert. As shown in **Figure 2**. Korla has a warm climate, sufficient light, and abundant water, heat, and soil resources, providing suitable growth conditions for long staple cotton that loves warmth and light. It is an important high-quality cotton producing area in China. The cotton planting area accounts for more than 90% of the total planting area of local crops.

### 2.2 Data collection and processing

#### 2.2.1 Ground survey data collection of aphid infestation

This study was carried out in the cotton field of Korla Experimental Station of the Plant Protection Research Institute of the Chinese Academy of Agricultural Sciences in Korla, Xinjiang. The tested cotton was sown in mid to late April, spot sown on the film. The cotton aphid stress naturally occurred in the field. Cotton aphids in Korla generally migrate during seedling emergence and boll period. The field experiments was performed during July 3rd-11th, 2018, when the cotton was in boll period, the peak occurrence period of cotton aphids in the study area. At this time, cotton aphids migrate in summer, causing significant decline in cotton yield in Xinjiang. The ground data were investigated to obtain the hyperspectral data of the cotton canopy and judge the infestation grade of aphids. The ground aphid infestation grade was judged according to the aphids on the back of cotton leaves and the characteristics of the leaves. The cotton leaves damaged by aphids shrink to the back, and the leaves grow deformed. The honeydew secreted by aphids causes the leaves to show oily light. The classification standards of cotton aphid infestation degree refer to the Chinese national standard (GB/T 15799-2011), see **Table 1**. According to the classification standards, the aphid infestation in the study area was divided into five grades, and a total of 60 ground sample points were selected, including 20 healthy cotton sample points, 20 grade 1 aphid infestation sample points, 10 grade 2 aphid infestation sample points, 6 grade 3 aphid infestation sample points, and 4 grade 4 aphid infestation sample points, to ensure the uniform distribution of sample points. Trimble geoexplorer 6000 series equipment was used to connect RTK terminals, the Chihiro location service was used to record the GPS information of each sample point, and the error was less than 2 cm.

**Table 1:** Severity grading standards for cotton aphids.

Severity of aphid infestation	Standard
Y0	No aphids on the leaves, and the leaves are flat
Y1	There are a few aphids on the leaves, but the leaves are not infested
Y2	There are some aphids on the leaves and the most severely infested leaves are wrinkled or slightly rolled, nearly semicircle
Y3	The leaves have more aphids and the most severely infested leaves are curled up to half a circle or even more
Y4	The leaves are heavily infested with aphids, and the most severely infested leaves are completely curled and spherical

#### 2.2.2 Ground spectrum acquisition and processing

The experimental period was the boll stage of cotton, when the cotton field was basically closed, which was conducive to the acquisition of cotton spectral information. The Field Spec Handheld portable non-imaging spectrometer was used to collect hyperspectral data of the cotton canopy. The wavelength range of the spectrometer is 325 nm-1075 nm and the spectral resolution is 1.4 nm. The spectral acquisition was carried out in sunny, windless, cloudless and well-lit weather. The acquisition time was between 10:00-16:00 Beijing time. The range of each sampling point was 4 m^2^, and the five-point sampling method was used to collect spectral data five times. Each measurement generated 10 spectral data, that is, the collection of ground spectral data of one sample point was completed. The collection of cotton canopy spectral data of 60 sampling points was completed according to this standard.

**Figure 3:**
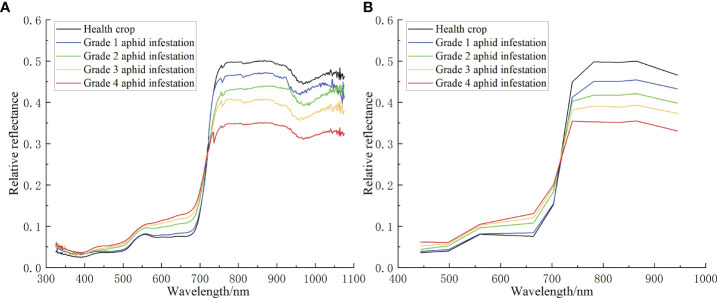
**(A)** The original ASD hyperspectral curve of aphid infestation at different grades in the cotton canopy. **(B)** The simulated ASD hyperspectral resampling curve of aphid infestation at different grades in the cotton canopy.

The abnormal spectrum was removed from the cotton canopy hyperspectral data collected at each sampling point, and the average hyperspectral data of each sampling point were obtained. All the obtained hyperspectral data were classified according to different aphid infestation grades, and then the average value was calculated. Finally, the average spectrum of aphid infestation at different grades of cotton was obtained, as shown in **Figure 3A**)

In this study, the Sentinel-2B spectral response function was used to resample the ASD hyperspectral data to obtain the Sentinel-2B simulated spectrum of the ground sampling point. The resampling formula is:


(1) $$R=\int_{r\_\lambda 1}^{r\_\lambda n}f(r)dr$$

where R is the reflectivity of a channel of the simulated wideband sensor; r is the relative reflection value of the wavelength of the narrow band sensor; *r*_*λn* and are the band range corresponding to the ith band of the broadband sensor; and *f*(*r*) is the spectral response function corresponding to the broadband sensor (Sentinel-2B in this study).

The specific operation method was to establish the average spectral data of each sampling point as a spectral library file in ENVI, and then resample the spectral library using the Sentinel-2B spectral response function of ENVI software to convert the narrow band spectrum into a wide band spectrum. The spectrum after resampling is shown in **Figure 3B**).

#### 2.2.3 Sentinel-2 satellite image processing

Sentinel-2 satellite data are from the ESA’s official website (https://scihub.copernicus.eu/), including two satellites A and B, which can cover 13 spectral bands, from visible near-infrared to short wave infrared, with different spatial resolutions. The two satellites complement each other, and the revisit period is 5 days. Sentinel-2 is the only data with three bands in the red edge range, which is very effective for monitoring vegetation health information. The images used in this paper are Sentinel-2B L1C data. The specific parameters are shown in **Table 2** below.

**Table 2:** Spectral characteristics of Sentinel-2B from the European Space Agency.

Sentinel-2B band	Center wavelength (μm)	Spatial resolution (m)
Band 1 - Coastal aerosol	442.3	60
Band 2 - Blue	492.1	10
Band 3 - Green	559.0	10
Band 4 - Red	665.0	10
Band 5 - Vegetation Red Edge	703.8	20
Band 6 - Vegetation Red Edge	739.1	20
Band 7 - Vegetation Red Edge	779.7	20
Band 8 - NIR	833.0	10
Band 8A - Narrow NIR	864.0	20
Band 9 - Water vapour	943.2	60
Band 10 - SWIR - Cirrus	1376.9	60
Band 11 - SWIR	1610.4	20
Band 12 - SWIR	2185.7	20

Sentinel-2B L1C is an atmospheric apparent reflectance product with geometric fine correction. Therefore, it is necessary to conduct radiometric calibration and atmospheric correction, convert the data product into surface reflectance and eliminate the influence of clouds on surface reflectance. The Sentinel-2B L1C products were processed using Sen2cor software. Since the study area is composed of two images, it is necessary to splice the remote sensing images, and then subset the remote sensing images through the vector map of the study area.

Because the spatial resolution of Sentinel-2B is different in different bands, the cubic convolution algorithm was used to resample the resolution of all bands to 10 m. To be consistent with the ASD spectral range (325 nm~1075 nm), ten bands of B1, B2, B3, B4, B5, B6, B7, B8, B8A and B9 were selected for the satellite band, and the spectral range was 442.3 nm-943.2 nm.

According to the spectral acquisition time of the cotton ground canopy, the Sentinel-2B image data of two scenes on June 3 and July 13 were selected. Among them, the satellite image on June 3 is the auxiliary data. The cotton planting area was extracted from the multitemporal satellite data, and the satellite image on July 13 is the corresponding time data collected by the ground spectrum for processing and analysis.

### 2.3 Method

#### 2.3.1 Extraction of cotton areas

**Figure 4:**
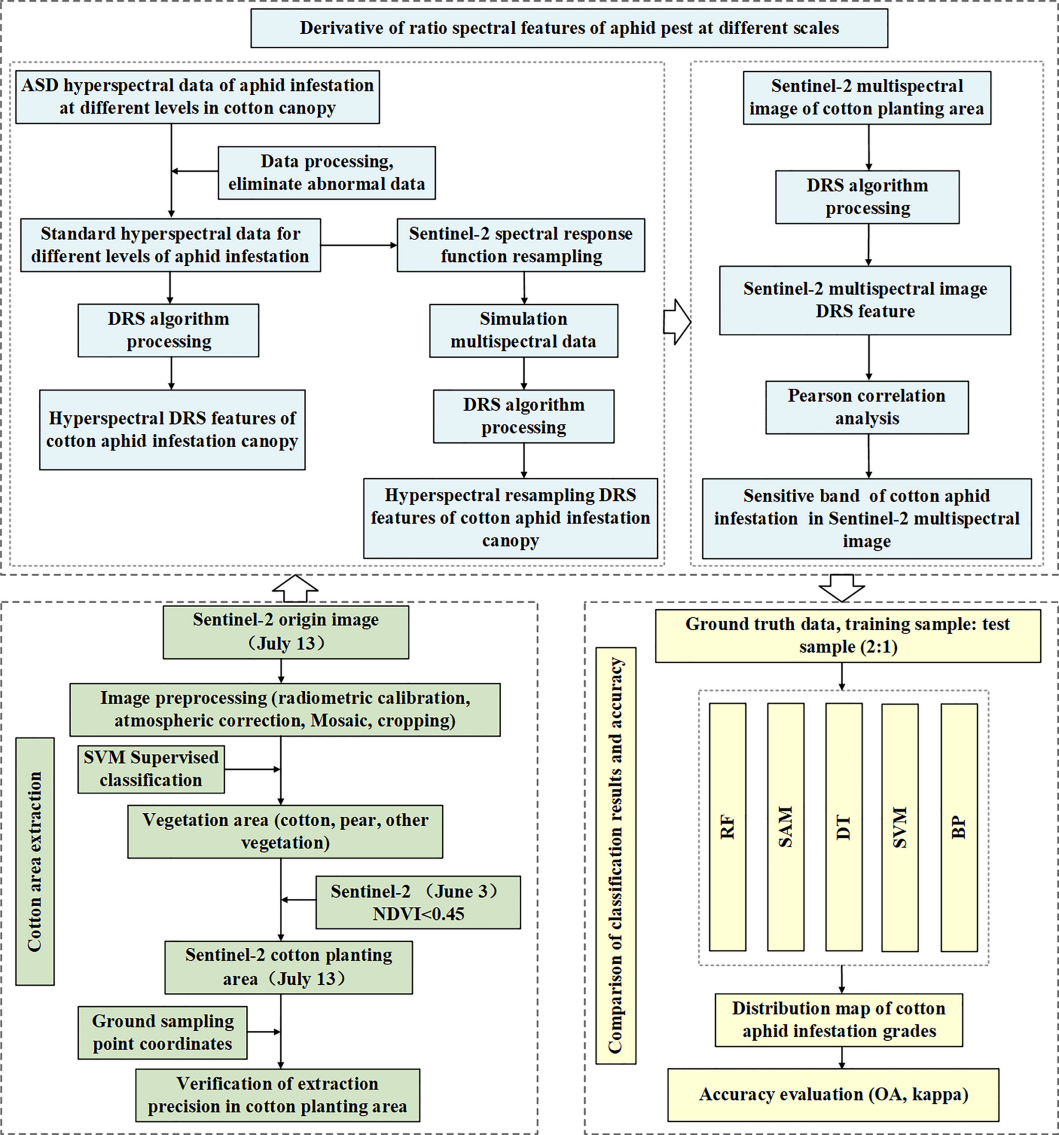
Flowchart of cotton aphid infestation classification at different grades based on Sentinel-2 multispectral images.

To more accurately study the spectral feature extraction algorithm of different grades of cotton aphid infestation and avoid the interference of other ground objects, it is necessary to extract the cotton planting area in the study area. First, vegetated and non-vegetated areas need to be separated, according to the feature types on the satellite images of the study area, five ground types, including vegetation, buildings, roads, bare lands and clouds, were selected as supervised classification samples. Then, the SVM classification algorithm was used to classify the Sentinel-2 images in the study area on July 13. According to the classification results, vegetation areas were extracted and made into masks. As the main vegetation in the Korla region includes cotton and fragrant pear trees, according to the difference of NDVI values of cotton and fragrant pear, the Sentinel-2 image on June 3 was used to extract the fragrant pear planting area by using the VI threshold method (the NDVI threshold value is 0.45, the fragrant pear trees were larger than 0.45, and the cotton trees were smaller than 0.45) to make a mask. The cotton planting area mask in the study area was obtained by subtracting the fragrant pear mask extracted on June 3 from the vegetation area mask extracted on July 13. Finally, the mask was used to subset the Sentinel-2 image on July 13 to obtain the cotton planting area in the study area. The main process is shown in **Figure 4**. SVM is a classical supervised classification algorithm, which shows many unique advantages in solving small sample, nonlinear and high-dimensional pattern recognition. Therefore, this study selects SVM as the method to extract cotton planting areas.

#### 2.3.2 DRS algorithm

The DRS algorithm is a special spectral processing method based on the linear mixing model. The purpose of using the algorithm is to eliminate the influence of other features in multiple features to obtain the correspondence between the mixed spectra of the target feature and multiple features and finally extract the band that is sensitive to the spectral information of the target feature. First, the ratio of two continuous spectra is calculated band by band, and then the ratio spectrum is differentiated to obtain the DRS curve (Philpot, 1991). Assuming that the spectrum contains n components, the linear spectral mixing model can be expressed by formula (2).


(2) $$\gamma (\lambda_{i})=\sum_{j=1}^{n}F_{j}\times \gamma_{i}(\lambda_{i})+\xi (\lambda_{i})$$

where i=1, 2, 3, …, m is the spectral band, j=1, 2, 3, …, n is the endmember component, and *F_j_* is the proportion of each endmember in the mixed pixel. Under the premise of neglecting the error term, assuming that there are only two substances in the mixed pixel, the linear spectral mixing model can be simplified into formula (3).


(3) $$\gamma (\lambda )=F_{1}\times \gamma_{1}(\lambda )+F_{2}\times \gamma_{2}(\lambda )$$

Both sides of formula (3) are divided by the spectrum of the second component at the same time, which becomes formula (4).


(4) $$\frac{\gamma (\lambda )}{\gamma_{2}(\lambda )}=F_{2}+\frac{F_{1}\times \gamma_{1}(\lambda )}{\gamma_{2}(\lambda )}$$

Derive from both sides of formula (4) to obtain formula (5).


(5) $$\frac{d}{d\lambda }\left(\frac{\gamma (\lambda )}{\gamma_{2}(\lambda )}\right)=F_{1}\times \frac{d}{d\lambda }\left(\frac{\gamma_{1}(\lambda )}{\gamma_{2}(\lambda )}\right)$$

It can be seen from formula (5) that the derivative spectrum is already independent of the content of the second component *F_2_*, that is, the spectrum after derivation is only linearly related to the abundance of the first component, but not to the abundance of the material component as a divisor.

In this study, each pixel in the Sentinel-2 satellite multispectral image can be considered a mixed pixel of background plant and pest-stressed plant canopy spectra. Among them, the background plants are relatively healthy cotton plants that are not affected by pests, and aphid infestation is the main stress. To remove the interference of the background healthy plant information, the healthy cotton spectrum was selected as the background endmember, and the Sentinel-2 imaging spectral data were subjected to DRS processing pixel by pixel with it as the denominator, so as to obtain the spectral features that only reflect the stress degree of the cotton aphid, and then the effective band that can reflect the cotton aphid infestation was extracted.

#### 2.3.3 RF classification algorithm

RF is an integrated machine learning algorithm based on multiple decision trees proposed by Leo Breiman (Breiman, 2001), which is suitable for solving high-dimensional nonlinear classification problems, can handle a large number of input variables and effectively avoids overfitting. It has good accuracy, generalization and robustness in the classification process (Rodriguez-Galiano et al., 2012). The RF algorithm uses the bootstrap sampling technique to randomly sample the input sample data with put back to form the data training sample set, and the remaining samples are collectively called Out of Bag Data (OOB), which can be used to test the model fitting accuracy. The decision trees corresponding to the sample subsets are constructed according to the feature splitting rules, and finally, the RF classification results are obtained by combining all the decision tree classification results through the simple voting method (Gu et al., 2016). The main flow of the algorithm is as follows:

(1) Suppose that the original sample set has N training samples, each sample has M features, and in the process of model training, n (n<N) sample subsets are extracted from the training samples with replacement through the bootstrap method.

(2) The decision tree model is constructed by using N training samples extracted each time and randomly selecting m (m<M) features at each node. The information of each feature is used to calculate and determine the growth direction of the decision tree at the split node.

(3) After K rounds of training, the results of each decision tree form a base evaluator sequence and then use a simple voting method to determine the results of the integrated evaluator.


(6) $$H(x)=arg\;max_{y}\sum_{i=1}^{k}I(h_{i}(X)=Y$$

where *H*(*x*) represents the final classification result of the model; *I*(.) is a property function; *h_i_* is a single decision tree classifier (base evaluator); and Y is the output target variable (target variable). The upper bound of the generalization error is:


(7) $$PE^{*}\leq \frac{\bar{\rho }(1-S^{2})}{S^{2}}$$

where PE* is the model generalization error; $\bar{\text{ρ}}$ is the correlation between decision trees; and S is the classification strength of the decision tree.

#### 2.3.4 Model construction and accuracy evaluation

In this study, the cotton planting area was extracted based on the SVM supervised classification and the VI threshold method through multitemporal Sentinel-2 data. Then, the DRS was processed on the cotton aphid spectrum at three scales, and the differences in spectral characteristics were analyzed and compared, which proved the effectiveness of the proposed algorithm. The Pearson correlation analysis algorithm was used to extract the bands significantly related to aphid infestation. Finally, combined with the RF classification algorithm, the multispectral image data samples after DRS processing were divided into training samples and test samples according to the ratio of 2:1 for model training. According to the model result, the Sentinel-2 image was classified into aphid severity grades, and the classification accuracy was calculated. To prove the superiority of this algorithm, we compared it with four commonly used classification algorithms. The confusion matrix algorithm was used as the evaluation standard of the stability and prediction ability of the constructed model, and the effect of the DRS algorithm was evaluated. The main process of this study is shown in **Figure 4**.

The confusion matrix, also known as the error matrix, is a standard format for accuracy evaluation in the form of a matrix with n rows and n columns. The specific evaluation indexes include four kinds: overall accuracy (OA), producer’s accuracy (PA), user’s accuracy (UA) and kappa coefficient, which reflect the accuracy of image classification from different aspects (Lewis and Brown, 2001; Luque et al., 2019).

Assuming that n represents the total number of samples in the classification accuracy evaluation, K represents the total number of classification categories in the classification accuracy evaluation, n_ii_ represents the number of samples correctly classified, and n_ij_ represents that they belong to class j and are wrongly classified into class i, the evaluation index formula is as follows:


(8) $$\text{OA}=\frac{\sum^{}n_{ii}}{n}$$

(9) $$\text{PA}=\frac{\sum^{}n_{jj}}{\sum^{}n_{ij}}$$

(10) $$\text{UA}=\frac{\sum^{}n_{ii}}{\sum^{}n_{ij}}$$

(11) $$\text{K}=\frac{n\sum_{i=1}^{k}n_{ii}-\sum_{i=1}^{k}\sum^{}n_{ij}\sum^{}n_{ij}}{n^{2}-\sum_{i=1}^{k}\sum^{}n_{ij}\sum^{}n_{ij}}$$

## 3 Results

### 3.1 Extraction results and precision evaluation of cotton planting areas

**Figure 5:**
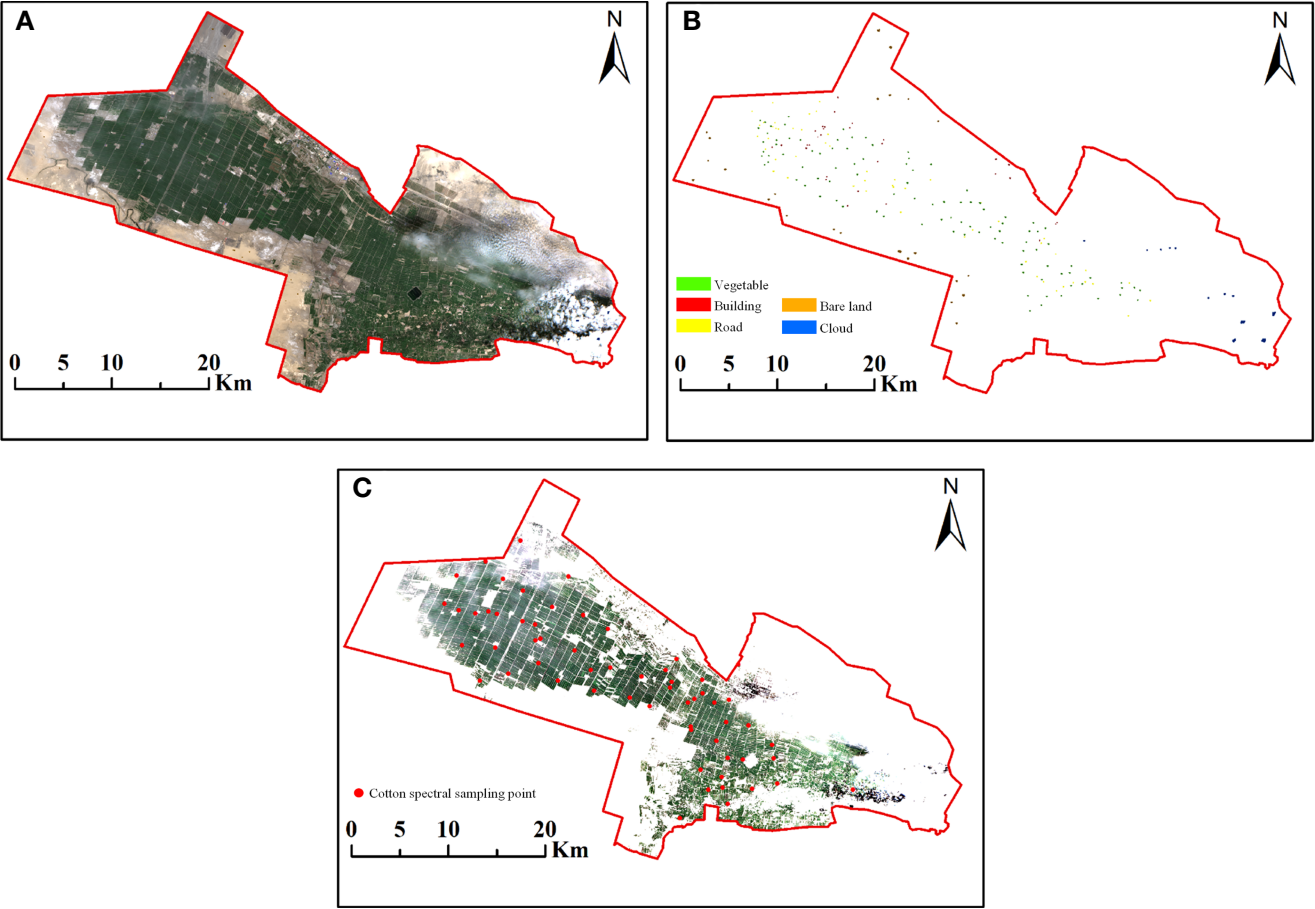
Sentinel-2 true color map, supervised classification sample map and cotton planting area extraction map of the study area. **(A)** True color map of Sentinel-2 multispectral image with red, green and blue bands corresponding to 4, 3 and 2 bands, respectively. **(B)** Supervised classification sample map of the study area, including five categories: vegetation, buildings, roads, bare lands, clouds. **(C)** Cotton planting area extraction results from Sentinel-2 multispectral images in the study area and location of cotton spectral sampling points.

Using Sentinel-2 images of the study area, the cotton planting areas extracted by a combination of SVM supervised classification algorithm as well as VI thresholding method are shown in **Figure 5C**). And the red points are the sample points of cotton spectral field collection (60 sample points), which are used to verify the accuracy of cotton planting area extraction. **Figure 5A**) shows the Sentinel-2 true color map of the study area, with the red, green, and blue bands corresponding to the 4, 3 and 2 bands of the Sentinel-2 image, respectively. **Figure 5B**) shows the sample map used for supervised classification in the study area. The samples were divided into five major categories based on feature: vegetation, buildings, roads, bare lands, and clouds, and the sample edges were bolded by 0.5 times for display purposes. It could be seen from **Figure 5C**) that the cotton planting area has been accurately extracted, and the background features such as buildings, roads, bare lands and clouds have been effectively removed. The accuracy of the cotton planting areas extracted results was verified by the longitude and latitude coordinates of the ground sample data in the study area. The results showed that 60 ground sample points were within the extracted planting areas, which showed the effectiveness of the extraction results, and laid the foundation for the next cotton aphid infestation severity classification.

### 3.2 Spectral feature analysis of cotton aphid infestation at different scales based on the DRS algorithm

#### 3.2.1 Ground ASD hyperspectral spectral features

**Figure 6:**
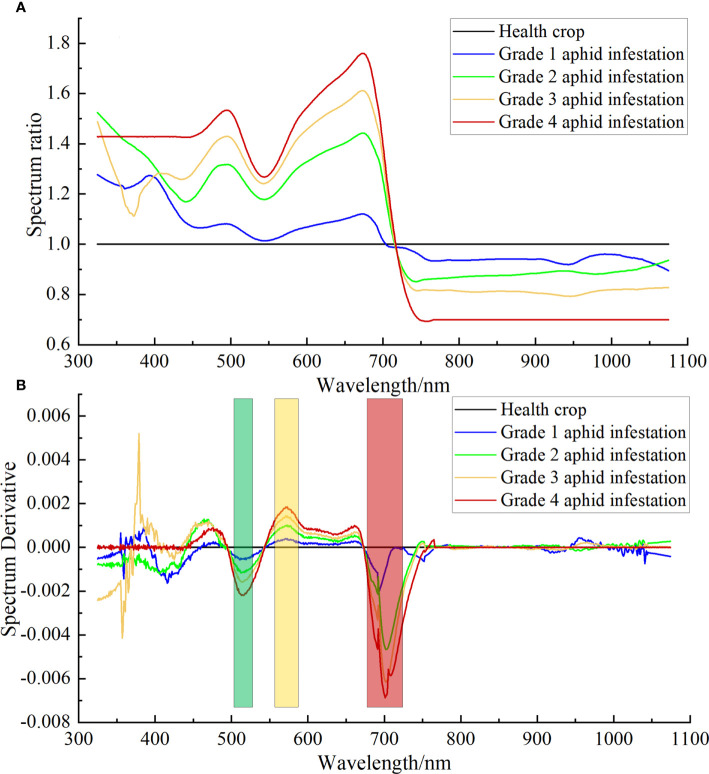
DRS results of ASD hyperspectral of cotton aphid infestation at different grades. **(A)** Ratio spectra of cotton plants infested by different grades of aphids to healthy plants. **(B)** DRS of cotton plants infested by different grades of aphids to healthy plants.

According to formula (4) in subsection 2.3.2, the ground ASD hyperspectral cotton canopy aphid infestation spectra of different grades in **Figure 3A**) were processed by the ratio algorithm, with the healthy cotton plants spectral as the divisor and the aphid infestation stress spectra as the dividend, and the results are shown in **Figure 6A**). After the ratio algorithm treatment, the spectral features of cotton plants under aphid-stressed were highlighted, and the higher the aphid severity, the more obvious the spectral features, and the lower the aphid severity, the flatter the spectral features and tended to the healthy plant spectra.

The spectra in **Figure 6A**) were derived according to formula (5) to obtain the DRS shown in **Figure 6B**). As described in subsection 2.3.2, the spectrum after derivation is only linearly related to the abundance as a divisor, but not to the component as a divisor. **Figure 6B**) shows the DRS obtained by taking the healthy cotton plant spectrum as the divisor. At this time, the spectral curve is no longer related to healthy vegetation information, but only to vegetation stress information. As seen from the figure, the peaks of the ratio spectral curves for different aphid severities appear in the visible and red-edge regions commonly used to characterize vegetation spectral features, which are similar to the results of a large number of existing studies (Guo et al., 2021). With the higher aphid severity grade, the absolute values of the spectra after the DRS treatment are larger, and the peaks appear at the 513 nm, 573 nm and 701 nm bands.

#### 3.2.2 Ground ASD hyperspectral resampling spectral feature

**Figure 7:**
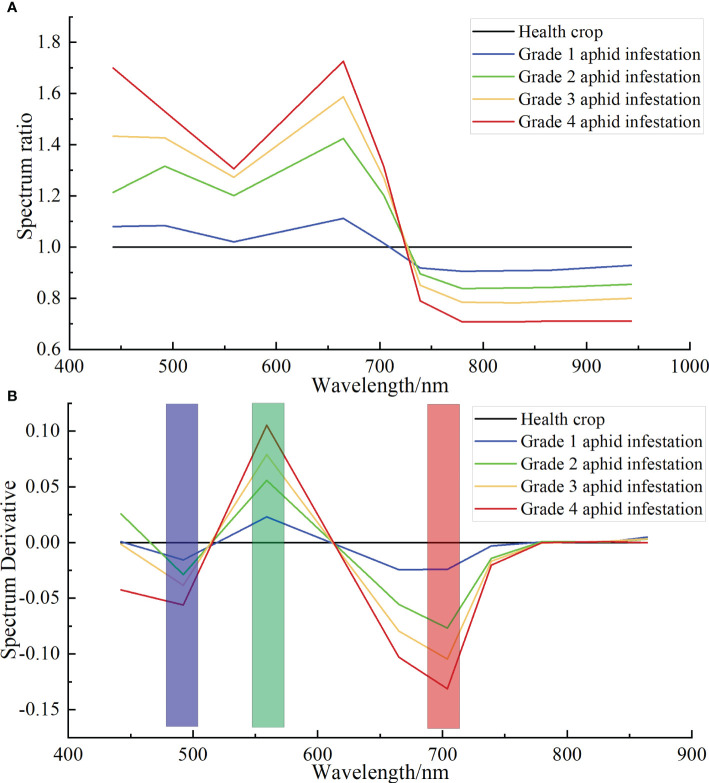
DRS results of ASD hyperspectral resampling of cotton aphid infestation at different grades. **(A)** Ratio spectra of cotton plants infested by different grades of aphids to healthy plants. **(B)** DRS of cotton plants infested by different grades of aphids to healthy plants.

Through formula (4), the spectral curves of cotton aphid infestation at different grades after ASD hyperspectral resampling were processed by the DRS algorithm. Similarly, using the healthy cotton plant spectra as the divisor and the aphid infestation stress spectra as the divisor (**Figure 7A**), the DRS results are shown in **Figure 7B**). From the results, it can be seen that in the spectral range of 442 nm to 943 nm, the waveforms of ASD resampled spectra are similar to those of the DRS results before ASD resampling, with three peaks in the blue, green and red-edge bands, and the peaks correspond to the bands of 492.1 nm, 559 nm, and 703.8 nm, respectively.

#### 3.2.3 Sentinel-2 multispectral imagery spectral features

**Figure 8:**
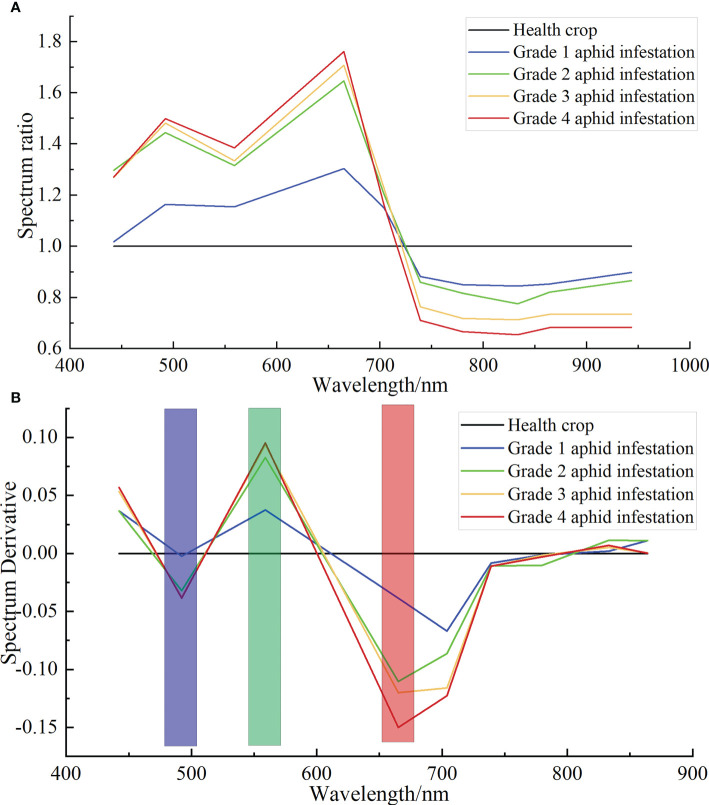
DRS results of Sentinel-2 multispectral of cotton aphid infestation at different grades. **(A)** Ratio spectra of cotton plants infested by different grades aphids to healthy plants. **(B)** DRS of cotton plants infested by different grades of aphids to healthy plants.

Based on the ground truth point coordinates of different grades of cotton aphid infestation, the multispectral data were extracted from the Sentinel-2 image, and then the average values were obtained to obtain the standard spectral curves of different grades of aphid infestation in the study area. Finally, taking the healthy cotton plant spectra as the divisor and the aphid infestation stress spectra as the divisor (**Figure 8A**), the DRS results of cotton aphid infestation based on Sentinel-2 multispectral data were obtained, as shown in (**Figure 8B**). The spectral characteristics of aphid infestation were significantly enhanced with the same three peaks, in which the peak bands corresponding to the grade 2, 3 and 4 aphid infestation were 492.1 nm, 559 nm and 665 nm, respectively, and the peak bands corresponding to the grade 1 aphid infestation were 492.1 nm, 559 nm and 703.8 nm, respectively.

#### 3.2.4 Spectral feature extraction of cotton aphid infestation

**Figure 9:**
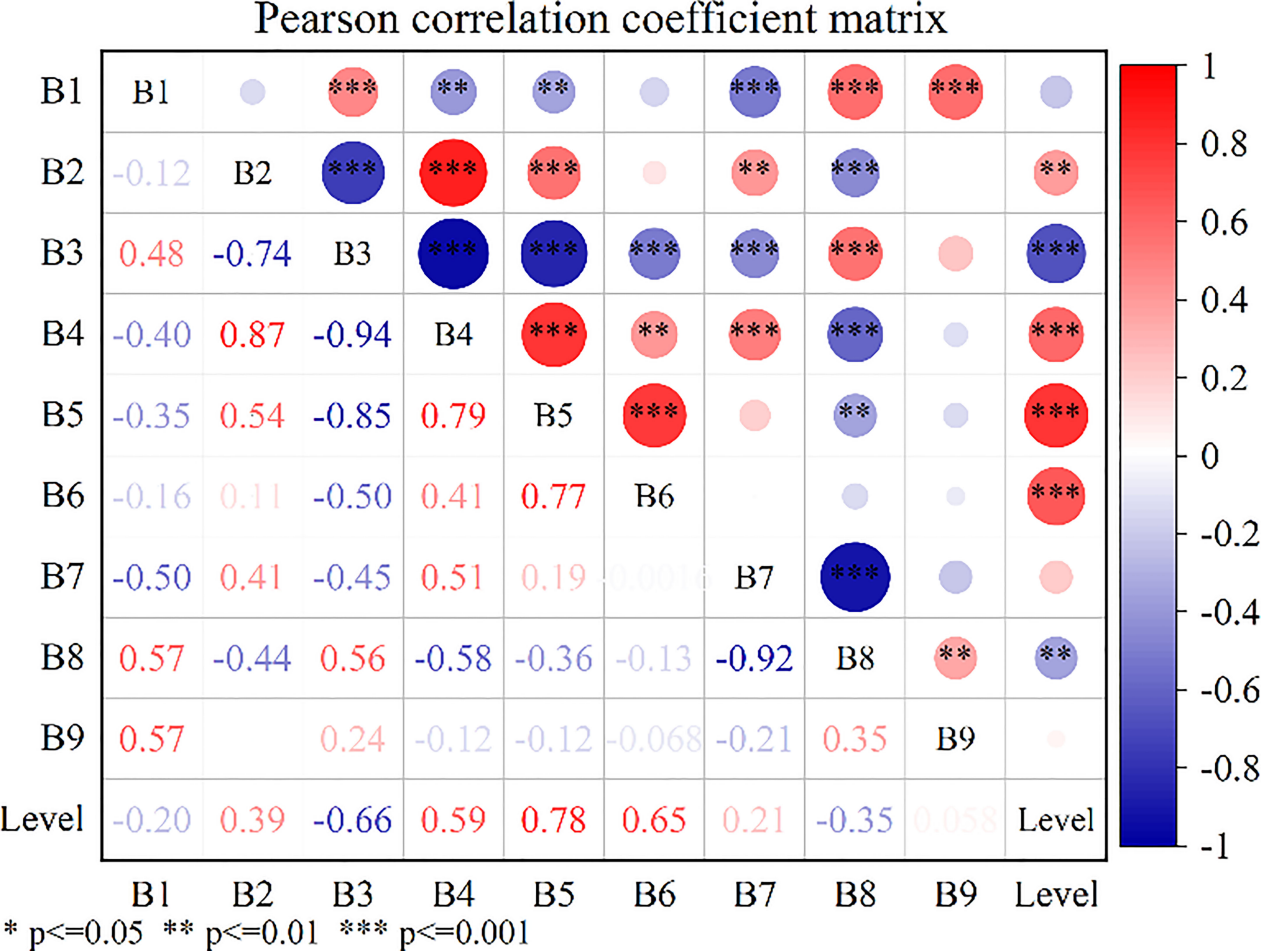
Pearson correlation analysis results of aphid infestation severity and DRS value in Sentinel-2 multispectral images. (* represents significant at the 0.05 level; ** represents significant at the 0.01 level; *** represents significant at the 0.001 level).

After DRS algorithm processing, there are nine band features remain in the Sentinel-2 satellite multispectral image, as shown in **Figure 8B**). To avoid the interference of unnecessary information, feature extraction was performed on the bands processed by the DRS algorithm to extract sensitive bands that are highly correlated with the severity of aphid infestation. Pearson’s correlation analysis was performed by using the aphid severity class values of 60 sample points with the DRS values of Sentinel-2 images, and the coefficient correlation plots were obtained as shown in **Figure 9**. The results showed that there was a positive correlation between the cotton aphid infestation grade and the spectral value treated by the DRS at the B2, B4, B5 and B6 bands, of which the B2 band was significantly correlated at 0.01 level, and the B4, B5, B6 bands were significantly correlated at 0.001 level. There was a negative correlation at bands B3 and B8, of which the B8 band was significantly correlated at 0.01 level, and the B3 band was significantly correlated at 0.001 level. There was no correlation at the B1 and B7 bands. When there is significant correlation between variables (in **Figure 9**, the “*” represents significant at the 0.05 level, the “**” represents significant at the 0.01 level, the “***” represents significant at the 0.001 level), it indicates a statistically significant correlation between the variables, the correlation coefficient R represents the closeness of the correlation. Generally, the R value between 0.2 and 0.4 indicates a general relationship, between 0.4 and 0.7 indicates a close relationship, and above 0.7 indicates a very close relationship. On this basis, B2, B3, B4, B5, B6, and B8 bands were selected as the input features of the random forest model.

### 3.3 Classification results and accuracy analysis based on the RF algorithm

**Figure 10:**
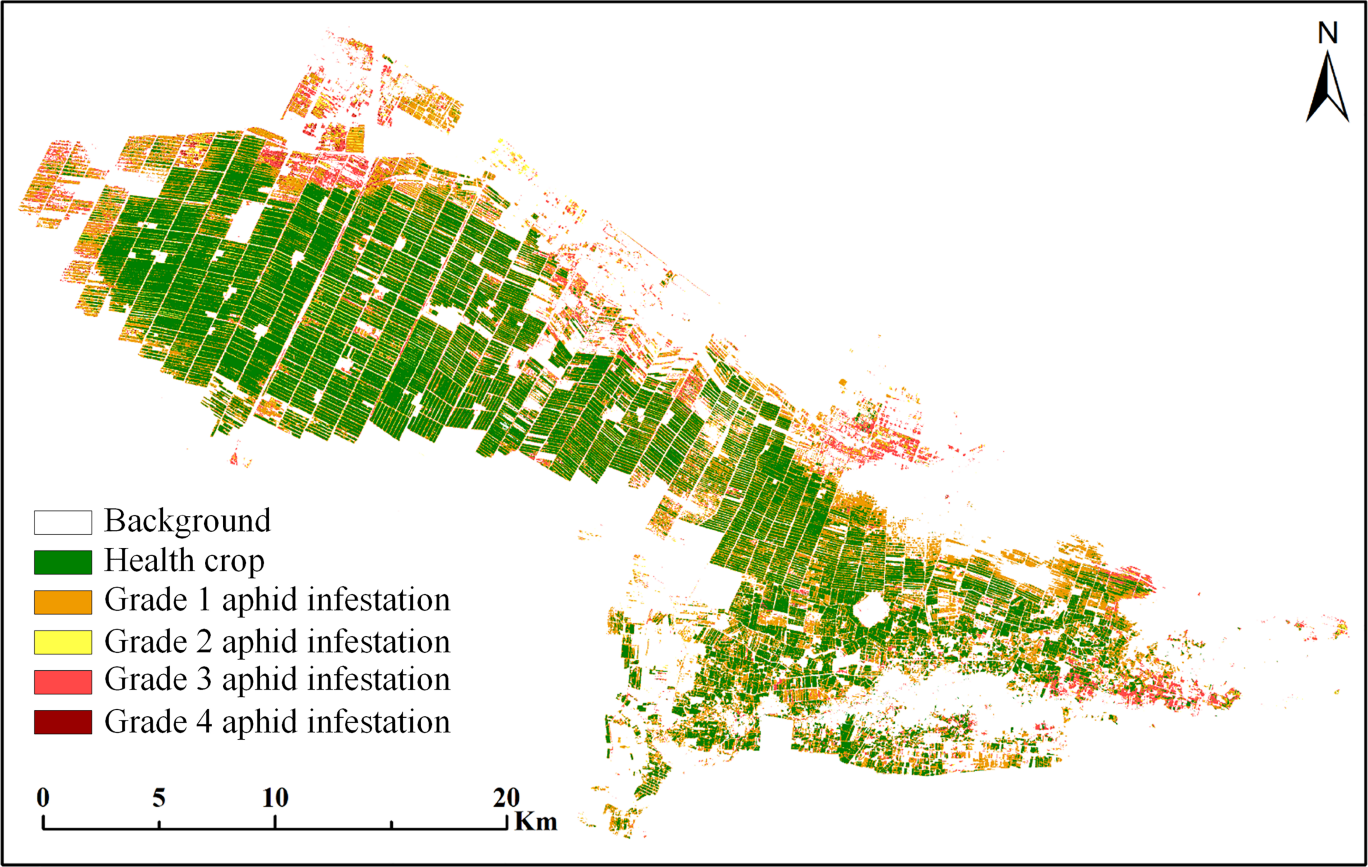
RF classification results of cotton aphid infestation at different grades.

Based on the extraction results of cotton planting areas in Section 3.1 and the results of DRS algorithm analysis and spectral feature selection in Section 3.2, the Sentinel-2 multispectral images were used to classify cotton pest severity at the regional scale by the RF algorithm model. First, 60 ground sample points were divided into a training group and a test group at a ratio of 2:1, including 40 samples for training and 20 samples for verification. Then, the RF algorithm model was trained by training samples, and the optimal model results were selected by the cross validation method to classify the severity of aphids in Sentinel-2 multispectral images. The pest classification standard was based on the severity of cotton aphids (**Table 1**), and the classification results are shown in **Figure 10**. It can be seen from the figure that there are great differences in the study area under aphid infestation stress. The areas with serious aphid infestation stress were mainly distributed at the edge of the study area, while other areas were less or not under aphid infestation stress, and most cotton was in a healthy state. The classification accuracy of the model is shown in **Table 3**. The OA is 80%, and the kappa coefficient is 0.73, thus obtaining excellent classification results.

**Table 3:** RF classification accuracy of cotton aphid infestation at different grades.

	Health	Grade 1	Grade 2	Grade 3	Grade 4	total	UA	OA	Kappa
Health	6	1				7	85.7%	80.0%	0.73
Grade 1		5	1	1		7	71.4%		
Grade 2			3			3	100.0%		
Grade 3				1	1	2	50.0%		
Grade 4					1	1	100.0%		
total	6	6	4	2	2				
Drawing accuracy	100%	83.3%	75%	50%	50%				

### 3.4 Results and precision comparison of different classification algorithms for cotton aphid infestation

**Figure 11:**
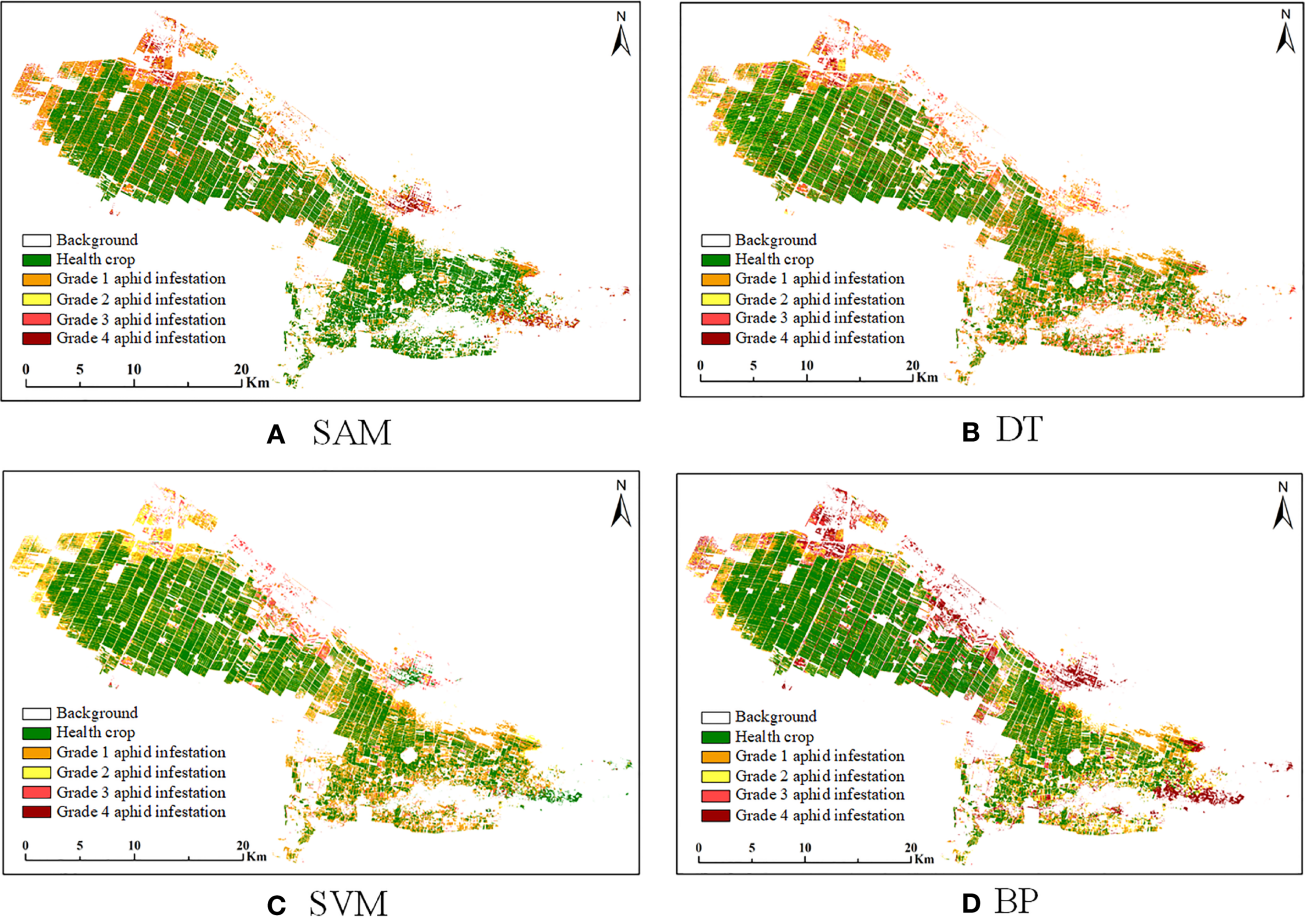
Classification results of four algorithms for cotton aphid infestation at different grades in the study area. **(A)** SAM, **(B)** DT, **(C)** SVM, **(D)** BP.

**Figure 12:**
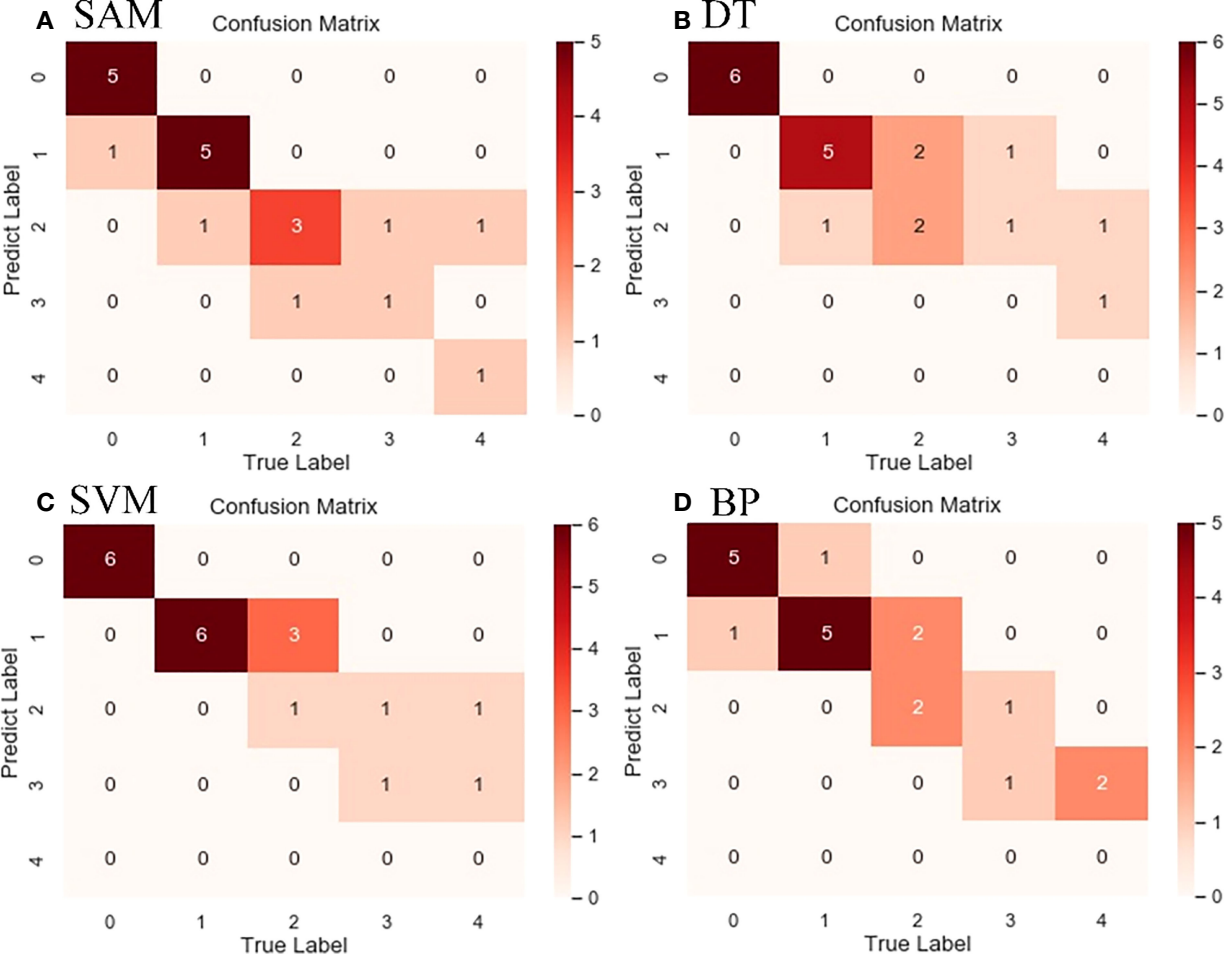
Confusion matrix of classification results of four algorithms for cotton aphid infestation at different grades in the study area. **(A)** SAM, **(B)** DT, **(C)** SVM, **(D)** BP.

To verify the superiority of the RF algorithm, four classical classification algorithms were selected to compare with the proposed algorithm. Among the four comparison algorithms selected, the SAM algorithm is a hyperspectral image analysis technology, which judges the similarity between the two spectral curve vectors by comparing the cosine between them and is commonly used in satellite hyperspectral image classification. The DT, SVM and BP algorithms are three commonly used machine learning classification algorithms. All four algorithms were performed in the same environment as the RF algorithm, and all of them were classified by the feature band selection results processed by the DRS algorithm. The highest accuracy was taken as the final classification accuracy using multiple cross-validation, and the classification results are shown in **Figure 11**. To facilitate comparison, the classification result accuracies of the four algorithms on the test dataset were presented in the form of a confusion matrix (**Figure 12**), and the overall classification accuracies and Kappa coefficients are shown in **Table 4**.

**Table 4:** Comparison of classification accuracy of four algorithms for cotton aphid infestation at different grades.

	SAM	DT	SVM	BP
OA	75%	65%	70%	65%
Kappa	0.67	0.52	0.59	0.53

From the direct comparison between the classification results of the four algorithms and the proposed algorithm, it can be seen that the classification results of the SAM, DT, SVM, BP and RF algorithms are not intuitively different, but there are differences in details. For example, the SVM algorithm has more classification for grade 2 aphids, and the BP algorithm has more classification for grade 4 aphids. The comparison of the accuracy evaluation results shows that the overall classification accuracies of the five algorithms, RF, SAM, SVM, DT and BP, are 80%, 75%, 65%, 70% and 65%, respectively, and the Kappa coefficients are 0.73, 0.67, 0.52, 0.59 and 0.53, respectively. From the comparison of the overall accuracy and Kappa coefficients, RF has the highest classification accuracy among the five algorithms, which proves the superiority of the algorithm proposed in this paper.

### 3.5 Effect evaluation of the DRS algorithm

**Figure 13:**
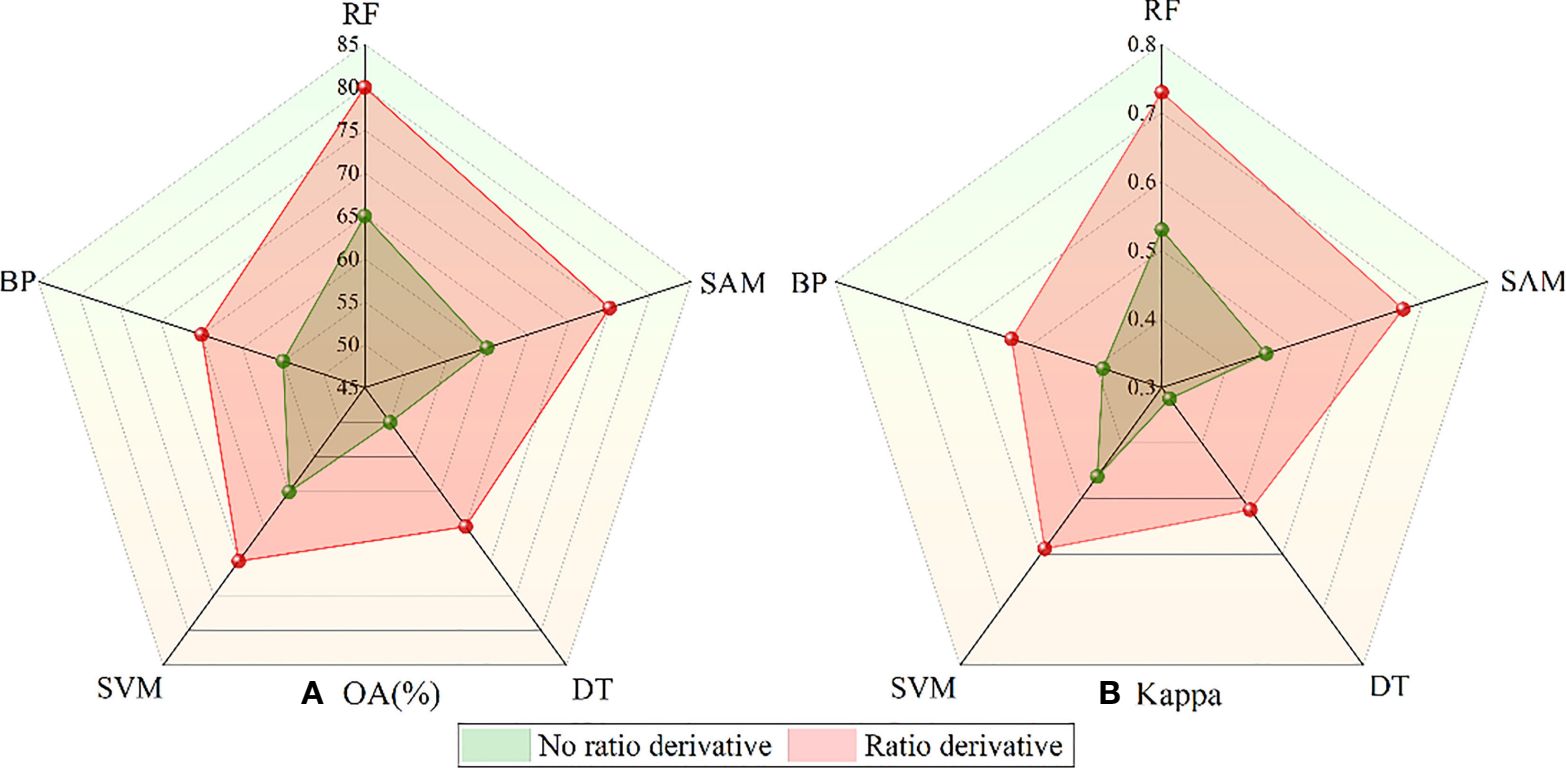
Accuracy comparison of different algorithms before and after processing by the DRS algorithm. **(A)** Comparison results of OA. **(B)** Comparison results of Kappa.

To verify the effect of the DRS algorithm on the classification accuracy, the Sentinel-2 original bands that have not been processed by the DRS algorithm were classified directly and compared with the final classification accuracy of the five algorithms proposed in this paper. The results are shown in **Figure 13**. Among the five classification algorithms, all obtained better performance after the DRS algorithm. The overall accuracy of the five algorithms increased by 13% on average, and the kappa coefficient increased by 0.176 on average. The overall accuracy of the RF algorithm was increased by 15%, and the kappa coefficient was increased by 0.2, which proves that the spectral features processed by the DRS algorithm can effectively improve the extraction accuracy.

### 3.6 Effect of VI features on classification accuracy

**Figure 14:**
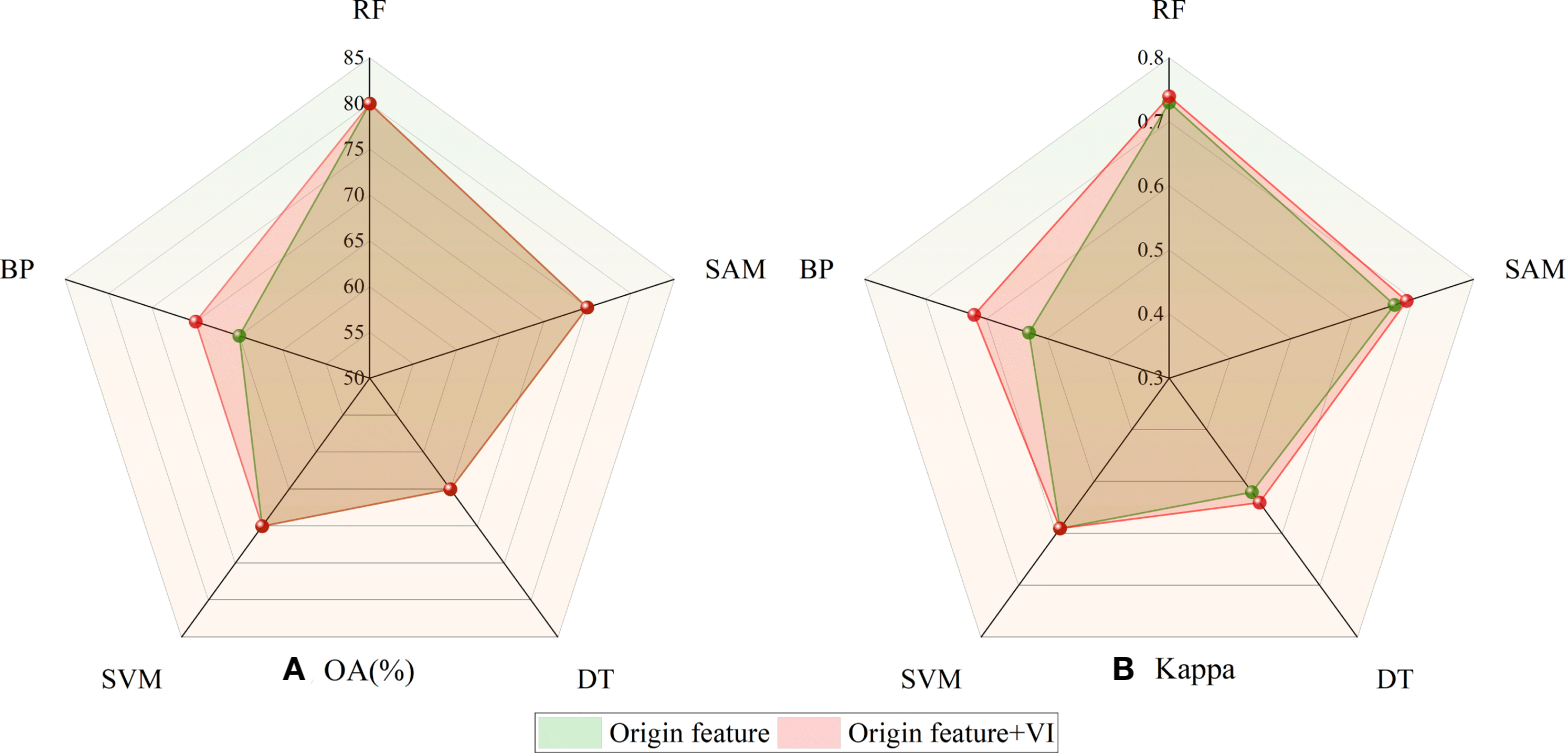
Accuracy comparison of different algorithms before and after adding VI features. **(A)** Comparison results of OA. **(B)** Comparison results of Kappa.

VI is a linear or nonlinear combination of visible and near-infrared wavelength reflectance based on the vegetation spectral features, to measure the state of surface vegetation and simplify spectral information (Zeng et al., 2022). In this section, ten vegetation indices related to crop pests were selected on the basis of original features, including normalized difference vegetation index (NDVI), green normalized difference vegetation index (GNDVI), triangular vegetation index (TVI), soil adjusted vegetation index (SAVI), atmospheric resistance vegetation index (ARVI), renormalization difference vegetation index (RDVI), enhanced vegetation index (EVI), red normalized difference vegetation index (NDVI705), modified red edge simple ratio index (mSR705), and modified red edge normalized difference vegetation index (mNDVI705), which were used to increase the features of the machine learning model and improve the classification accuracy. The classification accuracy of cotton aphid infestation before and after adding VI features is shown in **Figure 14**. From the OA and Kappa coefficients, it can be seen that the accuracy improvement after adding VI features is not obvious. The overall accuracy of the five algorithms increased by 1% on average, and the Kappa coefficient improved by 0.028 on average. The accuracy of BP algorithm is improved the most, which may be related to the model structure.

## 4 Discussion

The effectiveness of the DRS algorithm was demonstrated by the results of ground hyperspectral data processing, which lays a foundation for hyperspectral resampling and satellite multispectral data analysis. After the hyperspectral data were resampled by the Sentinel-2 spectral response function, although the number of bands and spectral information were greatly reduced, the key spectral features of pests were effectively retained, indicating that the spectral features of pests can also be characterized through limited bands, which lays a foundation for pest classification using satellite multispectral data. By comparing the DRS algorithm results of Sentinel-2 multispectral data with ground hyperspectral resampling, it could be found that the waveforms were roughly the same, and there were three peaks, which also confirms that the processed Sentinel-2 multispectral data can effectively retain the spectral information of pests, laying a foundation for the following classification. The threat of cotton aphids to cotton plants is mainly reflected in two aspects. On the one hand, it sucks the juice from the back or tender head of leaves, resulting in a decrease in leaf activity and a change in chlorophyll and water content, making the cotton leaves curl up towards the back. On the other hand, the excreta of Aphis gossypii easily breeds mould and affects leaf photosynthesis, which leads to spectral changes in the range of visible and near-infrared wavelengths with the increase of aphid infestation grade (Tang et al., 2022). Due to the limitation of satellite multispectral image resolution, although the influence of the background endmember can be effectively removed by DRS algorithm processing, there are still some uncertainty reasons that lead to some discrepancies. In the Sentinel-2 multispectral DRS results, the peaks of grade 2, 3 and 4 aphids at the red edge shifted to the visible part and appeared in the red band part, while the peak of grade 1 aphids remained unchanged.

It is a trend to monitor plant pests on a large regional scale by satellite multispectral images (Ruan et al., 2021). Based on the combination of the DRS algorithm and RF algorithm, this study used Sentinel-2 images to classify the severity of cotton aphids. It is important to point out potential areas for further improvements. The previous experiment used only the original feature of the Sentinel-2 image processed by the DRS. From the perspective of improving classification accuracy and referring to existing research, in addition to adding VI features related to pests in the model (Joalland et al., 2017), the texture features (Zhu et al., 2017) and mathematical statistical features (Vishnoi et al., 2022) will also be considered, which may have a certain impact on the classification accuracy. Therefore, it is necessary to study the fusion strategy of various features to improve the classification accuracy. In addition to feature extraction, the factors that affect the accuracy of pest classification are also related to the selection of classification methods. At present, machine learning algorithms have been applied in many fields and achieved good results (Lu et al., 2022; Maia et al., 2022). In this study, five machine learning algorithms were selected for comparison of classification accuracy, and the best classification performance was achieved by RF, demonstrating the good performance of the algorithm in terms of pest classification potential. Compared with traditional classification algorithms, RF performs well in terms of accuracy, robustness and computational requirements compared to traditional classification algorithms. At present, there are some new machine learning algorithms and deep learning algorithms that also perform well, which can be considered later. Due to the limitation of sample collection time, weather, sunlight and other factors, the number of sample points collected in this study was limited (60), especially only 4 sample points for grade 4 aphid infestation were obtained, which might have some influence on the accuracy of model evaluation results.

Xinjiang is the main cotton producing area in China, and the experimental station of the Institute of Plant Protection of the Chinese Academy of Agricultural Sciences in Korla, central Xinjiang, was selected as the study area. However, due to the differences in the natural environment and geographical location, there could be differences in cotton varieties and management measures in different cotton production areas, resulting in significant differences in pest characteristics, which might affect the accuracy of the proposed method. Therefore, it needs to be further tested in other study areas to verify its effects of monitoring pest grades in large scale areas, which should be taken into account in subsequent research. The present research only considers cotton aphid infestation, which is consistent with the conditions of the experimental area at that time. However, different insect pests may occur simultaneously in some areas, and even diseases may also occur simultaneously. The spectral characteristics of stress caused by different diseases and insect pests are sometimes very similar, and whether they can be distinguished by the present method remains to be further explored, which is also a difficult point in the current crop disease and pest research.

## 5 Conclusions

In this study, a classification method of cotton aphid severity based on the combination of the DRS algorithm and RF algorithm is proposed, and the results show that the method has great application potential. The DRS algorithm can effectively remove the interference of the pixel background endmember of Sentinel-2 multispectral satellite images, enhance the spectral features of cotton aphids at different grades. On the basis of feature extraction, five machine learning algorithms were used to classify aphid infestations. The results show that the RF algorithm has the highest classification accuracy, which proves the superiority of the RF algorithm. Combined with the DRS algorithm and the RF algorithm, using Sentinel-2 satellite multispectral data can realize the monitoring of cotton aphid infestation severity on a regional scale, and the accuracy can meet the actual needs, thus helping to guide the precise application of pesticides, control pest development, reduce production costs, and protect the ecological environment.

The method presented in this paper provides a new technology for future research, and may also be applicable to other crops. Future work could consider collecting a wider range of data from different regions to improve the algorithm. Compared with the rich spectral information contained in hyperspectral images, the spectral information of satellite multispectral images is weak. Therefore, it is necessary to enhance the spectral features related to pests through the DRS algorithm to improve the classification accuracy. At the same time, to avoid the interference of irrelevant information and data redundancy, it is also necessary to further improve the feature extraction algorithm and simplify the model as much as possible. At present, we only consider the DRS processing on the Sentinel-2 original bands to remove the impact of healthy cotton background on aphid classification in the pest spectrum. In the future, it is necessary to further analyze the impact of other parameters in the background spectrum on the inversion results. At the same time, it is considered to add pest characteristics to improve the applicability of the satellite multispectral platform in large-scale pest monitoring.

## Data availability statement

The original contributions presented in the study are included in the article/supplementary material. Further inquiries can be directed to the corresponding author.

## Author contributions

HF: Software, Formal analysis, Investigation, Methodology, Writing - original draft, Writing-review&editing. HZ: Software, Methodology, Conceptualization, Formal analysis, Writing - review & editing. RS: Formal analysis, Validation, Writing - review & editing. YY: Formal analysis, Resources, Writing - review & editing. ZL: Formal analysis, Validation, Resources. SZ: Resources, Writing - review & editing. All authors contributed to the article and approved the submitted version.

## Funding

This work is supported by the Fundamental Research Funds for the Central Universities (Grant No.2022JCCXDC01), the Yueqi Young Scholar of China University of Mining and Technology (Beijing) (Grant No.2020QN07), the Geological Research Project of the Hebei Bureau of Geology and Mineral Resources (Grant No.454-0601-YBN-DONH), and the State Key Laboratory of Coal Resources and Safe Mining Open Research Project [Grant No.SKLCRSM20KFA09], the National Natural Science Foundation of China (41701488).

## Conflict of interest

The authors declare that the research was conducted in the absence of any commercial or financial relationships that could be construed as a potential conflict of interest.

## Publisher’s note

All claims expressed in this article are solely those of the authors and do not necessarily represent those of their affiliated organizations, or those of the publisher, the editors and the reviewers. Any product that may be evaluated in this article, or claim that may be made by its manufacturer, is not guaranteed or endorsed by the publisher.
